# A conical passive magnetic bearing with constant stiffness

**DOI:** 10.1038/s41598-022-07988-6

**Published:** 2022-03-08

**Authors:** R. Bjørk, C. R. H. Bahl

**Affiliations:** Department of Energy Conversion and Storage, Technical University of Denmark - DTU, Anker Engelunds Vej, DK-2800 Kgs, Lyngby, Denmark

**Keywords:** Magnetic properties and materials, Mechanical engineering

## Abstract

A nested conical passive magnetic bearing is presented. The bearing consists of a nested conical rotor inside a conical stator, i.e. two coaxial tilted rings of permanent magnets, both with a rectangular cross section. Varying the cone or tilt angle of the rotor and stator we determine the rotor radius that provides the highest force for three different magnetization cases. For this optimal rotor radius, we show that the bearing with the highest volume normalized force also has the highest stiffness, and furthermore often also the highest varying stiffness with axial displacement. Finally, we show that a conical bearings with a tilt angle of $60^\circ$ has an almost constant stiffness and a linearly varying force with axial displacement, making it ideal as a bearing.

## Introduction

With an increasing focus on energy efficiency, there is a desire to identify and reduce any losses. For rotary machines such losses typically occur as friction at the bearings. Since the development of the strong rare earth based permanent magnets in the 1970’ies and 1980’ies^5^, these materials have been considered for magnetic bearings. The high magnetisation allows for a strong repulsive force between sets of opposing magnets, thus supplying the lift for even heavy rotating parts.

However, as proven by the British mathematician Samuel Earnshaw in 1842^11^ levitation based on non-diamagnetic materials can never be stable in all directions, except in cases where the bearing utilizes a gyroscopic effect^2,28^. Thus, a magnetic bearing based on the repulsion between permanent magnets is inherently unstable. In order to function, some degree of stability is required in the lift (often vertical) direction. This will result in instability in the radial direction and therefore some kind of stabilising, i.e. restoring, force is required in the radial direction. Such a stabilising bearing is referred to as an active magnetic bearing, while the permanent magnet assembly providing the lift is referred to as the passive bearing.

Initial modelling studies of the simplest type of passive magnetic bearing, namely two axially magnetised rings stacked on top of each other was done by Yonnet^37^. He found that the lifting strength was the same if the rings were radially magnetised. Similar results for the stiffness were later found semi-analytically by Ravaud *et* *al*.^26,27^ for solid rings of repulsive permanent magnets, and stacks of such systems have also been studied^33,38^. Closed form expressions for the lift has also been derived analytically^10,21^. Such systems have also been tested experimentally^19,25^.

A large number of papers have been published on different passive bearing configurations. The stiffness and lift in different situations have been studied both numerically and experimentally, often with a comparison to models, see e.g. Ref.^6^. To validate the models experimental setups for testing bearings both concentric and off-centre have been presented^7,24,30,39^. The design with the maximum radial stiffness has been found numerically in Ref.^22^, by considering the air gap between rotor and stator. Optimally, a bearing configuration should provide both lift and stability, and thus concepts have been suggested with magnet placements attempting to achieve this^24^. The dynamics of the active bearing has also been investigated^12,31,32^.

For all passive bearings, it is the stiffness of the radial component that determines the active force needed to keep the bearing stable. A lower negative value of this means a lower force and thus less required energy. However, in all existing studied bearings, the ideal balance between the provided lift and axial stability and the radial instability has not been reached. While bearings with low instability have been realized, the stiffness often varies with axial displacement, making it hard to realize an easily controllable active bearing.

In the current study we analyse a parameterised system designed to supply both lift and stability, by a nested concentric placement of two conical rings. The idea is that if the bearing is shaped like two nested conical rings, with the rotor inside the stator, the magnetic forces will both have a component providing a lifting force along the axis of the bearing, but also a component along the radial direction, which might allow the stiffness along this direction to be designed as desired.

The properties of a conically nested passive magnetic bearing have not previously been considered in literature. The properties of a number of concentric bearings stacked in a cone-shape has been considered^1^, but here the tilting of the concentric magnetic rings were not considered, i.e. the bearing was not truly cone-shaped. A number of cone-shaped active magnetic bearings have been considered^13,18,20^, including complex structures with cone-shaped teeth^35^ and bearing for use in magnetically suspended flywheels^9,34^. However, all of these works considers the dynamics of the active bearing and not the properties of a nested conical passive bearing.

The bearing investigated here is a passive bearing and will thus always be unstable in one direction, as per Earnshaws theorem, ignoring dynamic effects. The bearing will thus have to be stabilized by an active magnetic bearing, as is always the case for a passive magnetic bearing^19^.

Here the forces in the nested conical bearing are found through modelling using the finite element framework Comsol Multiphysics, which is applicable as the bearing is axi-symmetric. This means that the modelling can be performed at high resolution. A modeling approach is justified, as the bearing modelled is a fully magnetostatic system for which simulated magnetic fields are know to fully reproduce experimentally measured fields, see e.g. Refs.^4,8,14,16,23,29^. The specific model used here has previously been used to calculate the force of passive magnetic bearings and compared with experiments with good agreement^24^. For these reasons, the results of the modeling work presented here are directly applicable to practical real-world passive magnetic bearings.

## Physics

We consider an axi-symmetric bearing which provides a force in the *z*-direction, $F_\mathrm {z}$, which we also denote as the “lift”. Besides its lift, a bearing is also characterized by its stiffness, ${\mathbf {K}}$, which is the negative of the derivative of the force in a specific direction with respect to the displacement in the same direction, i.e.

1 $$\begin{aligned} {\mathbf {K}}= & -\left( \frac{dF_x}{dx}{\hat{x}}+\frac{dF_y}{dy}{\hat{y}}+\frac{dF_z}{dz}{\hat{z}}\right) \nonumber \\= & -\left( K_x{\hat{x}}+K_y{\hat{y}}+K_z{\hat{z}}\right) . \end{aligned}$$

The fact that passive magnetic levitation is not possible is summed up in Earnshaw’s theorem^11^, which states that the sum of the three stiffness components is equal to zero. Here we consider a bearing with a radial symmetry where thus $K_x = K_y = K_r$. This means that the radial stiffness in the following can be calculated from the stiffness in the *z*-direction as

2 $$\begin{aligned} K_r = -\frac{1}{2}K_z. \end{aligned}$$

as long as the bearing is axi-symmetric.

To determine the force and stiffness of the bearing considered, we have realized a magnetostatic finite element (FEM) axi-symmetric model of the bearing in Comsol Multiphysics. The equation solved in the FEM framework is the magnetic scalar potential equation

3 $$\begin{aligned} -\nabla \cdot (\mu _{0}\mu _{r}\nabla V_\mathrm {m})=0~. \end{aligned}$$

Here $\mu _{0}$ is the permeability of free space, $\mu _{r}$ is the relative permeability, which is assumed to be constant and isotropic, and $V_\mathrm {m}$ is the magnetic scalar potential. The magnetic field is then calculated as $-\nabla V_\mathrm {m} = {\mathbf {H}}$.

To determine the force on either the stator or the rotor, Maxwell’s stress tensor, ${\mathbb {T}}$, must be integrated across the surface covering either of these^11^

4 $$\begin{aligned} {\mathbf {F}}=\oint _{S'}{\mathbb {T}}\cdot \mathrm {d}{\mathbf {a}} \end{aligned}$$

where $S'$ denotes the closed surface surrounding one of the two bearing parts and $\mathrm {d}{\mathbf {a}}$ is an area element of this. With no electrical fields, Maxwell’s stress tensor is given by

5 $$\begin{aligned} T_{ij}=\frac{1}{\mu _0}\left( B_iB_j-\frac{1}{2}\delta _{ij}B^2\right) , \end{aligned}$$

where ${\mathbf {B}}=(B_x,B_y,B_z)$ is the magnetic flux density. This integration is automatically done in the Comsol framework.

As both the rotor and stator are axi-symmetric, there is no angular dependence on the force. This also means that the force on the rotor will be the same regardless of the rotation speed of the rotor.

The built-in Comsol Multiphysics solver *GMRES*, which is a Generalized Minimum RESidual iterative method solver, is used to solve Eq. (3) on the finite element mesh. The computational volume is chosen large enough that the boundaries of the simulation volume do not affect the calculations and a fine enough mesh is chosen that the results are verified not to vary when further increasing the mesh size. As we consider an axi-symmetric model, the simulations are 2D, and a very high mesh resolution can be used, eliminating the known convergence issues with bearing finite element models in 3D^6^. As the system modelled is a magnetostatic system, the finite element modeling approach is known to fully reproduce the field created by experimental setups. Therefore a pure modeling is justified.

**Figure 1:**
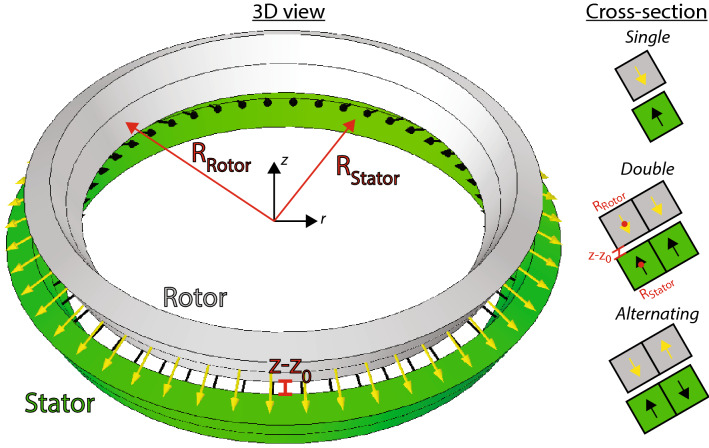
The bearing geometry considered. The stator, shown in green, and the rotor, shown in grey, each consists of two tilted rings of permanent magnets with a quadratic cross-section. The rotor has an inwards magnetization normal to the surface of the rings, while the stator has an outwards magnetization, as indicated by the arrows. The radii $R_\mathrm {rotor}$ and $R_\mathrm {stator}$, which are the radius to the center of the lower ring in either, is also indicated, as is $z-z_0$. The rotor is displaced along the *z*-axis. The bearing illustrated here has a tilt angle of $30^\circ$, as also illustrated in Fig. 2. On the right is shown a cross-section of the bearing, with the three types of magnet configurations considered, as explained in the text. The geometry illustrated on the left is the double bearing.

**Figure 2:**
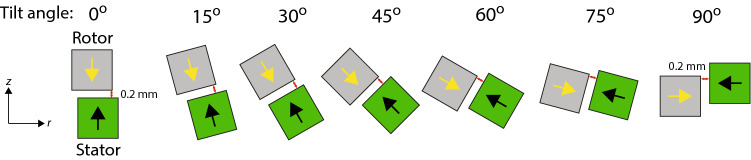
A cross-section of the bearing for the different tilt angles of the rings. The black and yellow arrows indicated the direction of magnetization of the rotor and stator, respectively. Shown as a red line is the minimum distance between the rotor and the stator in each configuration, which is always 0.2 mm.

## Bearing geometry

We consider a bearing consisting of nested conical axi-symmetric magnet rings with a quadratic, i.e. square, cross-section. This bearing concept is illustrated in Fig. 1 and can be said to resembles two nested magnetic “bowls”. We consider a design where the stator is larger than the rotor, i.e. the rotor is nested inside the stator. The rotor and stator are centered on the same axis, and when load is applied on the rotor bearing this will move along the *z*-axis. The stator has a magnetization direction that is along the inwards normal to its surface, while the rotor has an outwards magnetization direction normal to its surface. Thus, there is repulsion between the rings. Switching both magnetization directions results in an identical situation. It is noted that while producing such tilted conical rings of permanents magnets can be difficult if the rings are to be one piece, the rings can easily be made of cubes of permanent magnets placed in a ring, similar to Ref.^24^.

In order to ensure that there is always a clearance between the rotor and the stator, we define the value of $z_0$ to be the *z*-value at which the stator and rotor have a minimum distance between them of 0.2 mm as shown in Fig. 2. The conical rings that make up both the rotor and the stator have quadratic cross-section side lengths of 3 mm. The radius of the center of the tilted ring in the stator is fixed at 24 mm, while it is varied for the rotor. As shown in Fig. 2 we consider different tilt angles of the stator and rotor rings in the bearing, and these are always tilted by the same angle. The tilt angle is varied continuously, and as the magnetization is always normal to the surface of the magnets to allow for use a standard cube magnets, the passive bearing will at a tilt angle of $90^\circ$ end up being a repulsive-type radially magnetized bearing^38^.

We also consider extended ring geometries, with twice the cross-section and different magnetization directions, as shown in Fig. 1, where the rotor and stator radii are also indicated. The variation of cross-section is done because the ratio of the magnets’ dimensions (cross-sectional width or height) to the length of the air-gap is known to have a major influence on the bearing stiffness^17^. The three different magnetization directions considered are the single bearing, which consists of two tilted concentric rings with opposite magnetization, the double bearing which is similar to the single bearing, except with two magnets in each tilted ring, and finally the alternating bearing is similar to the double bearing, except that the magnetization of one part of the magnet is reversed in both the stator and rotor rings. We assume that the permanent magnets in the bearing have a remanence of 1.4 T and a relative permeability of $\mu _\mathrm {r} = 1$, which is very close to the typical experimental values for NdFeB magnets^15^.

## Results

All results presented in this manuscript are directly available from the data repository in Ref.^3^.

Initially, a variation study was conducted to determine the optimal geometry of a bearing as function of the tilt angle and the bearing type. Tilt angles from $0^\circ$ to $90^\circ$ in steps of $15^\circ$ were considered. Combined with the three bearing types studied, this gives a total of 21 bearings modelled.

For each of these 21 bearings the radius of the rotor, $R_\mathrm {rotor}$, was varied from 19 mm to a value such that the minimum separation was 0.2 mm between the stator and the rotor in steps of 0.5 mm and the relative *z*-position, $z-z_0$ in 8 steps from 0 to 3 mm. For a tilt angle of $0^\circ$ where the gap is always at least 0.2 mm, the maximum $R_\mathrm {rotor}$ was 26 mm. It is noted that at $z-z_0=0$ mm the minimum distance between the rotor and the stator is 0.2 mm. This makes $z_0$ a function of $R_\mathrm {rotor}$, i.e. the rotor radius can be larger than the stator radius, as long as the rotor is vertically positioned higher than the status. The $z-z_0=0$ mm ensures that the minimum distance between the rotor and the stator is always kept and that these never overlap.

When the rotor radius is varied the volume of it changes. To account for this, in the following we consider the force in the *z*-direction normalized with respect to the total volume of the magnet rings in both the stator and the rotor.

**Figure 3:**
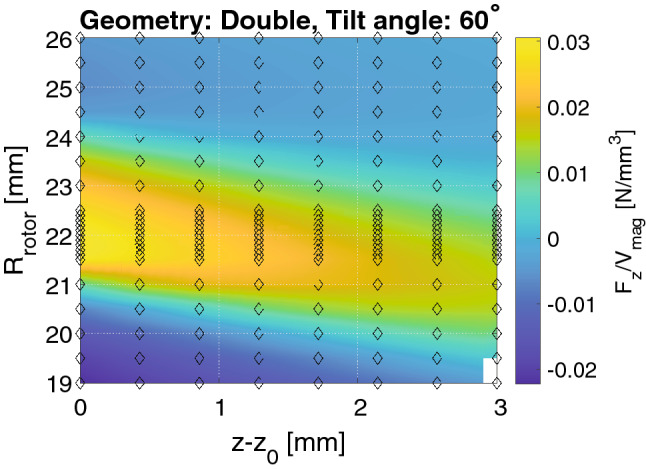
A surface map of the volume normalized force, $F_\mathrm {z}/V_\mathrm {mag}$, as function of the radius of the rotor and the relative *z*-displacement for the bearing with a double geometry and a tilt angle of $60^\circ$. The diamonds show the data points used to interpolate the surface. Note that $z-z_0$ is a function of $R_\mathrm {rotor}$ and thus the absolute $z-$position of the rotor increase as the rotor radius increases. At high values of $R_\mathrm {rotor}$ the rotor is entirely above the stator.

An adaptive routine is used to determine the optimal rotor radius to a precision of 0.1 mm. First, the volume normalized force was computed in steps of 0.5 mm for $R_\mathrm {rotor}$, and afterwards with 0.1 mm steps between the three highest values at $z-z_0=0$ mm. Figure 3 illustrates this by showing the volume normalized force, $F_\mathrm {z}/V_\mathrm {mag}$, as a function of the rotor radius and the $z-z_0$-displacement of the rotor for a bearing with a double geometry and a tilt angle of $60^\circ$.

**Figure 4:**
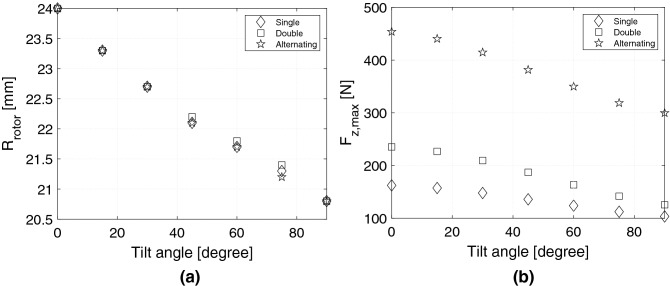
(**a**) The rotor radius, $R_\mathrm {rotor}$, at which the volume normalized force, $F_\mathrm {z}/V_\mathrm {mag}$, at $z-z_0=0$ mm is largest, (**b**) the largest force at the optimal rotor radius, for the different bearing geometries and tilt angles considered.

For all bearings the value of $R_\mathrm {rotor}$ where the volume normalized force is largest at $z-z_0=0$ mm are shown in Fig. 4, i.e. for the different tilt angles and bearing configurations. To compute these values, it is not necessary to perform a variation in *z*, i.e. calculate values different from $z-z_0=0$ mm, but this is nevertheless done in Fig. 3 to acquire an understanding of the variation of the bearing with $z-z_0$ for all values of $R_\mathrm {rotor}$. As can be seen from Fig. 4 the optimal value of $R_\mathrm {rotor}$ is almost identical for the different bearing geometries considered, with decreasing rotor radius for increasing tilt angle, as expected. The maximum force are also shown at the optimal value of $R_\mathrm {rotor}$ in Fig. 4.

**Figure 5:**
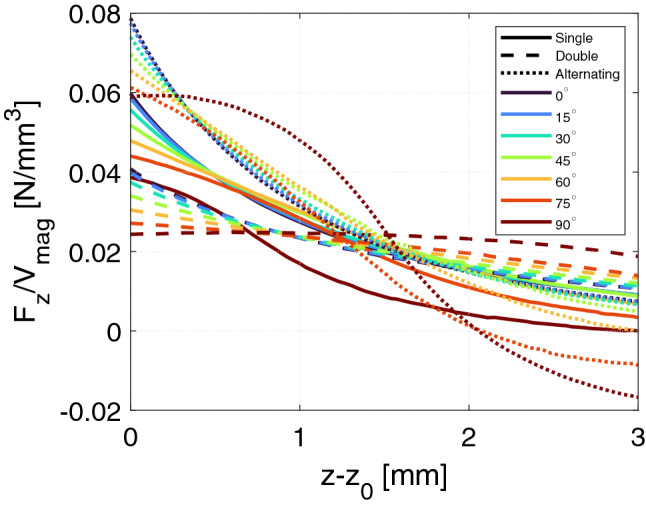
The volume normalized force in the *z*-direction as function of the relative displacement in the *z*-direction for the different bearings considered.

For each for the optimal $R_\mathrm {rotor}$ values the volume normalized force was computed at $z-z_0$ values from 0 to 3 mm in 80 steps to obtain a high resolution data set. The results are shown in Fig. 5. As can be seen from the figure, the alternating bearing has the largest volume normalized force, followed by the single bearing and finally the double bearing, regardless of tilt angle. This clearly shows that for the tilted bearing considered here, adding additional rings of permanent magnet, as is the case going from the single to the double bearing, does not result in a gain in volume normalized force. However, if one part of the ring is flipped, it can be an advantage. For all configurations the larger the tilt angle, the lower the volume normalized force.

Notice that for high $z-z_0$ values the force can become negative for the Alternating geometry. This is because the magnets in this geometry have opposite magnetization direction and at such a high $z-z_0$ the configuration is such that the rotor and stator magnets attract each other.

**Figure 6:**
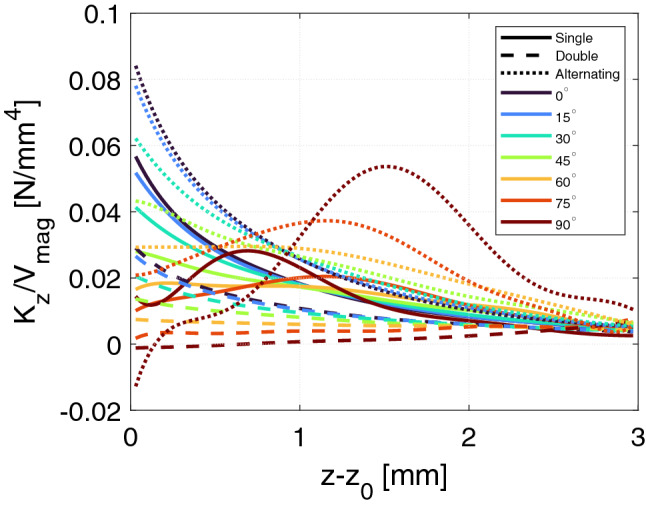
The volume normalized stiffness in the *z*-direction as function of the relative *z*-displacement for the different bearings considered. The stiffness is the *r*-direction can simply be calculated according to Eq. (2).

For each volume normalized force curve, we fitted a double exponential function of the form $F_\mathrm {z}/V_\mathrm {mag}=ae^{b(z-z_0)}+ce^{d(z-z_0)}$ and from this fit calculated the stiffness, $K_\mathrm {z}$, as the derivative with respect to *z*. This was done to smooth out small numerical variations in the force calculations, which can lead to large variations in the computed stiffness. The volume normalized stiffness in the $z-$direction is shown in Fig. 6. The $r-$direction stiffness can be calculated using Eq. (2) but is not shown due to brevity. The results follow the same trend as the volume normalized force shown in Fig. 5, i.e. that the lower tilt angles result in the largest absolute stiffness, and that the alternating design is the type with the largest absolute stiffness.

**Figure 7:**
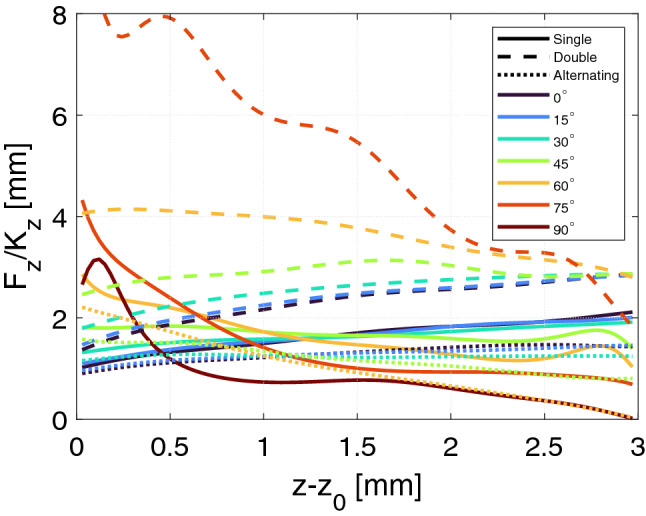
The ratio of the axial force of the bearing to its axial stiffness, as a function of the relative *z*-displacement for the different bearings considered.

We can also directly compare the force provided by the bearing and the axial stiffness of the bearing. Shown in Fig. 7 is the ratio of the axial force provided by the bearing to its axial stiffness. As can be seen from the figure, the double bearing generally has the highest ratio. Interestingly the trend as a function of tilt angle changes as a function of relative $z-$displacement, with the high tilt angles having the highest ratio at lowest relative $z-$displacement, but the lowest ratio at high displacements.

Typically the desired properties of a bearing are that it provides a high amount of lift at a relative low absolute stiffness, such that the required force from the active bearing is as small as possible. Of course a relative low stiffness will mean a high displacement with varying load, so the choice of stiffness is always a compromise. An initial idea to improve the performance of the bearing is to scale it up. As the magnetic field scales with the geometry of the bearing, the absolute lift of the bearing will increase as the size of the bearing is increased. However, the relative stiffness, which is the gradient of the force with respect to the size of the bearing remains constant.

**Figure 8:**
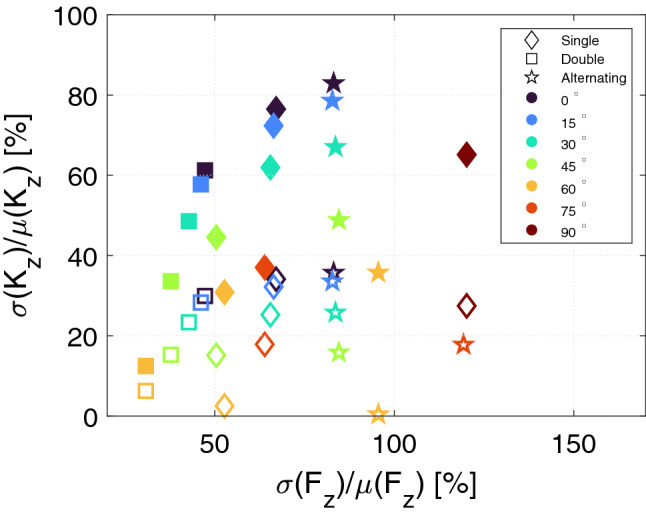
The relative standard deviation of the stiffness in the *z*-direction in percent as function of the relative standard deviation of the force in the *z*-direction in percent. The filled symbols consider the full $z-z_0$-range from 0 to 3 mm, while the unfilled symbols consider only the $z-z_0$-range from 0 to 1 mm. Only bearings which are stable in the *z*-direction, i.e. have $K_\mathrm {z}>0$ over the *z*-range considered, are shown.

This argument shows that scaling the bearing can be used to provide more lift, but it does not change the stiffness of the bearing. Therefore it is crucial to consider more in-depth the stability of the bearings simulated here. Considering this, we determine the bearing which would be easiest to control by an active bearing over the *z*-range considered here. This would be a bearing that has a constant $K_\mathrm {z}$-value in the *z*-range considered, but which also provides a high force over this range. This can be investigated by considering the relative standard deviation, $\sigma /\mu$, i.e. the standard deviation, $\sigma$, divided by the mean, $\mu$, for both the force and stiffness over the *z*-range considered. This is shown in Fig. 8 for all bearings which have $K_\mathrm {z}>0$ over the *z*-range considered, i.e. which are stable in the *z*-direction. It is noted that $\mu$ now denotes the mean, and is not to be confused with its earlier usage as the relative permeability.

Choosing the best bearing from Fig. 8 depends on the desired characteristics of the bearing. If one desired a bearing that is easy to control, i.e. has a mostly constant stiffness, the double geometry with a tilt angle between $60^\circ$–$75^\circ$ is clearly the best. However, as shown in Fig. 5 this bearing also provides the lowest lift of the bearings considered. Depending on the active bearing used to stabilize the passive bearing, it is possible that it was the maximum negative stiffness in the movement range considered that was important to dimension the active bearing. In this case passive bearings with a tilt angle of $45^\circ$ seem to provide a good compromise between lift and stiffness.

When a smaller $z-z_0$ range of 1 mm is considered, all bearings with a tilt angle of $60^\circ$ display almost constant stiffness as a function of displacement. This is a remarkable property, as such a bearing is very easy to control with an active bearing. Furthermore, the alternating bearing with a tilt angle of $60^\circ$ has a high value of $\sigma (F_\mathrm {z})/\mu (F_\mathrm {z})$, which means that it has a linear variation in *z*, with a large force at $z-z_0=0$ mm and a low force at $z-z_0=1$ mm. This is actually an ideal bearing, as the exact load on the rotor is not of importance, as long as it is between the minimum and maximum lift, and at the same time the stiffness is constant regardless of the load.

It is noted that the $0^\circ$ and $90^\circ$ for the single and double configurations is similar to the classical bearing geometry presented in Refs.^21,26,36,37^. Indeed, the $0^\circ$ has the highest relative force. The range of the relative stiffness, however, is larger, allowing more freedom in the design. Thus, by varying the tilt angle and the bearing geometry it is possible to tailor a bearing to the desired characteristics.

## Potential improvements

Additional methods to improve the force and stiffness of the presented bearings may also be considered. One option is to consider the shape and also exact magnetization direction of the two opposing rings in the single configuration. Only the doubling in one direction has been studied here, but other changes could be considered. Varying the exact direction of the magnetization in the rings could also be explored, to see if better configurations could be determined. Another option could be to look at closing the flux loop on the “back” side of both the rotor and stator rings in the alternating design with iron pieces. Previously, this has been shown to increase the stiffness and lift in a magnetic bearing^24^.

Finally, we remark that the conical bearing could also be reversed, such that the bearing provided an attractive force. In this configuration it could be used in an “upside-down” configuration to compensate gravity on the rotor by supplying a pull instead of a lift.

## Conclusion

We presented a concentric magnetic bearing consisting of two coaxial nested concentric rings of opposing permanent magnets as the rotor and stator. Both magnet rings had a rectangular cross-section. We examined three different configurations, and determined the rotor radius that provided the highest force as a function of the tilt angles of the stator and rotor rings.

For this optimal rotor radius, we showed that the bearing with the highest force also has the highest stiffness, and furthermore often also the highest variation in stiffness with axial displacement. Over a 1 mm displacement in *z*, a bearing with a tilt angle of $60^\circ$ has a constant stiffness and a linearly varying force, meaning that is can support a large variety of loads, making it the ideal bearing.

## Data Availability

All data presented in this manuscript are directly available from Ref.^3^. The simulation files are each typically $\sim 10$ Gb and are available upon request from the corresponding author.
